# Do We Still Need to Assess Post-procedural Radial Artery Compression Time and Radial Artery Occlusion in Patients Who Undergo Transradial Coronary Intervention?

**DOI:** 10.7759/cureus.35129

**Published:** 2023-02-17

**Authors:** Fawaz Bardooli, Dileep Kumar

**Affiliations:** 1 Interventional Cardiology, Mohammed Bin Khalifa Bin Sulman Al Khalifa Specialist Cardiac Centre, Awali, BHR; 2 Cardiology, Mohammed Bin Khalifa Bin Sulman Al Khalifa Specialist Cardiac Centre, Awali, BHR

**Keywords:** radial artery hematoma, trans-radial, radial artery compression time, radial artery occlusion, compression device

## Abstract

Background and objective

Transradial access (TRA) for interventional coronary procedures has now been widely accepted as the preferred vascular site approach. The duration of post-procedure compression has been shown to be a crucial factor and different hemostatic devices used in this regard have been compared. In this study, we aimed to compare the post-procedure compression time, radial artery occlusion (RAO), hematoma, and bleeding between the transradial (TR) band and AIR band for radial artery patency among patients presenting at a tertiary care hospital.

Methodology

This observational study was conducted at the Department of Cardiology of Mohammed Bin Khalifa Bin Sulman Al Khalifa Specialist Cardiac Centre, Awali, Bahrain from 06/03/2022 to 05/06/2022. The research involved patients of either gender who had a positive Barbeau test (type A to C) and were receiving percutaneous coronary intervention via a transradial route. Patients who underwent transradial coronary intervention were classified into two separate groups, depending on whether an AIR band (group A) or a TR band (group B) compression was used. Following coronary catheterization, radial hemostatic compression devices were used. The results were documented both during and after the hemostatic compression. The data were analyzed using IBM SPSS Statistics version 23 (IBM Corp., Armonk, NY).

Results

Of the total 100 patients included in the study, the majority were males (86%) and aged more than 50 years (83%). AIR band was successfully removed in 32 patients (64%) in less than four hours, compared to the TR band, which was removed in less than four hours in two patients (4%) only (p=0.001). The incidences of bleeding (p=0.790) and RAO (p=0.495) were similar between the AIR band group and the TR band group. Hematoma was not seen in any of the patients in either group.

Conclusion

AIR band was observed to be more efficacious in decreasing the radial artery compression time. However, the difference in RAO was insignificant in the short term, and follow-up studies are required to see if the AIR band is associated with any long-term benefits.

## Introduction

In the past few decades, transradial access (TRA) has been recognized as the optimum vascular site approach for percutaneous coronary intervention or coronary angiography worldwide [1]. Campeau performed coronary angiography with TRA in 1989, and Kiemeneij et al. described their radial percutaneous coronary intervention experience in a study published in 1995 [2,3]. Many researchers have indicated that TRA is associated with key benefits such as early ambulation, shorter hospital stay, lower procedural cost, as well as patient satisfaction, preference, and prognosis [1,4-5].

One of the major drawbacks of the TRA approach is radial artery occlusion (RAO), which can be symptomatic or asymptomatic [4,6]. The incidence of RAO reportedly ranges from 0.8% to 30% [6]. Factors like sheath size and long hemostasis time are responsible for RAO [7]. By utilizing a smaller sheath size and a compression device that shortens the time of hemostasis, RAO can be effectively prevented [6].

Various compression hemostatic devices have been used and found to be effective and safe in attaining hemostasis [1,4,8-9]. Terumo designed the TR band in Japan, and it is currently widely used in China to aid with radial artery hemostasis following transradial procedures [9]. The radial artery is compressed and hemostatic is attained by adding a certain amount of air into the TR band's balloon. With the variation in the size of the wrists of patients, a fixed air volume may produce variable pressures on wrists, resulting in ineffective compression [8,10].

The AIR band, on the other hand, is a recently developed radial compression device that aids in radial artery hemostasis following a TRA procedure. It's a latex-free, 26-cm-long, self-adhesive wristband with a bulb and clear window to help visualize the puncture site. The luer valve is linked to the clear fill tube's end, allowing any standard syringe to be used to deflate and inflate the bulb with air to compress the radial puncture site. AIR bands also provide superior patient comfort, and their adhesive nature ensures a secure fit around patients' wrists, preventing dislocation and movement [11].

To the best of our knowledge, this is the first study to compare the efficacy of the TR band with that of the AIR band. We have conducted this study to compare the post-procedure compression time, RAO, hematoma, and bleeding between the TR band and AIR band in patients following TRA procedures.

## Materials and methods

This cohort study was conducted at the Department of Cardiology of Mohammed Bin Khalifa Bin Sulman Al Khalifa Specialist Cardiac Centre, Awali, Bahrain from 06/03/2022 to 05/06/2022 after obtaining approval from the Ethical Review Committee (No: CTD-ij-2021-0033 on March 06/2022). A sample size of 100 patients, with 49≈50 in each group, was estimated using statistics of moderate to severe pain in the TR band as 22% [9] and in the new hemostatic compression device as 1.7% [9], with the power of test as 80%, and 95% confidence level. Patients undergoing percutaneous coronary intervention through transradial approach of either gender with age ≥18 years having a positive Barbeau test (type A to C) [7] were included in the study. Patients with bleeding diathesis or those on oral anticoagulation, those with acute infection, mental disorder, hemorrhagic disease, thrombocytopenia, uremia, and patients who refused to give consent were excluded from the study. A non-random consecutive sampling technique was applied for sample selection. Informed consent was obtained from all the eligible patients prior to data collection. Two independent parallel cohorts of patients who underwent transradial coronary intervention were included. The selection of the cohort was based on the compression band that was applied: either the AIR band (group A) or the TR band (group B). In the AIR band, 7 ml of air was inflated and in the TR band, 13-15 ml of air was inflated as per manufacturer guidelines. These bands were applied after removing the sheaths without any compression prior to placement. Patients in both groups were managed as per standard protocol in terms of antiplatelet and anticoagulation. Following coronary catheterization, radial hemostatic compression devices were used. The outcomes were documented both during and after the hemostatic compression. The outcome measures were time from band placement to complete removal, RAO, hematoma, or bleeding. Patients' baseline characteristics, demographic details, and contact information were also collected.

The data were analyzed using IBM SPSS Statistics version 23 (IBM Corp., Armonk, NY). Frequency and percentage were used to present data related to age groups, gender, hypertension, obesity, diabetes mellitus, smoking status, family history of ischemic heart disease, and outcomes. Chi-square/Fisher's exact test was applied to compare age groups, gender, hypertension, obesity, diabetes mellitus, smoking status, family history of ischemic heart disease, and outcomes between the TR band and AIR band. A p-value ≤0.05 was considered statistically significant.

## Results

Of the total 100 patients included in the study, the majority were males (86%) and aged more than 50 years (83%). There was a significant difference in terms of age (p=0.003) and gender (p=0.021) between the groups. Out of 100 patients, almost 64% were hypertensive, 53% were diabetic, 20% were obese, 12% were smokers, and 19% had a family history of ischemic heart disease. The proportion of patients with hypertension (p=0.001), diabetes (p=0.038), and family history of ischemic heart disease differed significantly between groups. Group-wise baseline characteristics are displayed in Table 1.

**Table 1:** Baseline characteristics of both groups of patients (n=100) *Statistically significant

Variables	Total (n=100), n (%)	Group A (n=50), n (%)	Group B (n=50), n (%)	P-value
Age group, years				
≤50	17 (17%)	3 (6%)	14 (28%)	0.003*
>50	83 (83%)	47 (94%)	36 (72%)	
Gender				
Male	86 (86%)	47 (94%)	39 (78%)	0.021*
Female	14 (14%)	3 (6%)	11 (22%)	
Hypertension				
Yes	64 (64%)	41 (82%)	23 (46%)	0.001*
No	36 (36%)	9 (18%)	27 (54%)	
Diabetes mellitus				
Yes	53 (53%)	33 (66%)	20 (40%)	0.009*
No	47 (47%)	17 (34%)	30 (60%)	
Smoker				
Yes	12 (12%)	4 (8%)	8 (16%)	0.218
No	53 (88.3%)	46 (92%)	42 (84%)	
Family history of ischemic heart disease				
Yes	19 (19%)	10 (20%)	3 (6%)	0.037*
No	81 (81%)	40 (80%)	47 (94%)	
Obesity				
Yes	12 (20%)	9 (18%)	10 (20%)	0.799
No	48 (80%)	41 (82%)	40 (80%)	

AIR band was successfully removed in 32 patients (64%) in less than four hours, compared to the TR band, which was removed in less than four hours in two patients (4%) only (p=0.001). In 20% of the patients, the AIR band was removed in four to five hours while it was removed in five to six hours in 16% of the patients. In the TR band group, 56% of the patients had their bands removed in four to five hours and 40% had the band removed in five to six hours. There was a significant difference in the time from band placement to complete removal between the groups (p=0.001).

The incidence of bleeding was similar between the AIR band and TR band (18% vs. 16%, p=0.790). Hematoma was not seen in any of the patients in either group. In the AIR band group, no incidence of RAO was observed, while two incidences of RAO were observed in the TR band group. However, there was a statistically insignificant difference in incidences of RAO between the groups (p=0.495) (Table 2).

**Table 2:** Comparison of outcomes between the groups (n=100) RAO: radial artery occlusion

Variables	Group A (n=50), n (%)	Group B (n=50), n (%)	P-value
Bleeding			
Yes	9 (18%)	8 (16%)	0.790
No	41 (82%)	42 (83.3%)	
Hematoma			
Yes	0	0	N/A
No	50 (100%)	50 (100%)	
RAO			
Yes	0	2 (4%)	0.495
No	30 (60%)	29 (58%)	

## Discussion

After a percutaneous coronary intervention, many types of radial hemostatic compression devices have been found to be efficacious, well-tolerated, and safe. Compression devices have been compared in the literature for local vascular problems, hemostasis, and patient comfort [9,12-14]. The TR band keeps the radial artery open during hemostasis, reducing the likelihood of RAO in the future, and it usually provides excellent patient comfort. Compression devices have a transparent structure and are used for selective compression and visual management of the radial artery in order to maintain patency and allow for blood to flow freely [9,10]. Whereas, the AIR band, which aids in radial artery hemostasis following a TRA procedure, is a latex-free, 26-cm-long, self-adhesive wrist band with a bulb and clear window to help visualize the puncture site. AIR bands also provide excellent patient comfort, and their adhesive nature ensures a secure fit around patients' wrists, preventing dislocation and movement [11].

The TR band has been found in numerous studies to be more effective in achieving hemostasis. The incidence of hematoma or hemorrhage during TR band radial compression has been reported to be 14-26% in the literature [10,15-17]. In the study by Wang et al., 14% of the patients had a hematoma in the TR band group while 12% of the patients had a hematoma in the new compression device group. They suggested that both compression devices were efficacious in attaining hemostasis. Rathore et al. evaluated the TR band and Radistop compression devices and found that both groups were identical in terms of major and minor hematoma (p>0.05) [10]. Malik et al. found that major and minor hematomas developed in 16% and 22% respectively of patients with TR band application, while only 8% of the patients with Prelude SYNC DISTAL radial compression device experienced complications. Thus, they concluded both techniques were efficacious in attaining hemostasis with a negligible time difference between the groups [9]. In the current study, both devices, i.e. AIR band and TR band, led to no cases of hematoma, with almost similar rates of minor bleeding between the two groups (18% vs. 16%, p=0.790).

Pancholy et al. found that merely two hours of compression following the removal of the arterial sheath significantly reduced the probability of RAO 24 hours later compared to six hours of compression (5.5% vs. 12%, p=0.025). Another randomized trial published in 2015 by Dharma et al. found that compression for more than four hours increased the incidence of RAO compared to compression for less than four hours (OR: 3.11, 95% CI: 1.665.82, p=0.001) [18]. In the current study, we observed a significant decrease in compression time with the AIR band as compared to the TR band. Almost 27% of patients had faster band removal (i.e., in three hours), but in the TR band group, none of the patients had effective band removal in three hours. Furthermore, RAO was found to be nonsignificantly associated with the type of band used for radial artery compression up to 24 hours in the AIR band and TR band groups (0% vs. 4%, p=0.495). Thus, shorter band removal time is not only important for patient ease or comfort, but also for a shorter hospital stay, lower incidence of RAO, and patient satisfaction. Hence, the AIR band is safer, more reliable, and more efficacious than the TR band in attaining patent hemostasis following radially accessed coronary catheterization procedures as it enables a significant decrease in compression time, which may lead to shorter nursing time and lower hospital expenses.

To the best of our knowledge, this is the first local observational study conducted to compare the post-procedure compression time, RAO, hematoma, and bleeding between the TR band and AIR band for radial artery patency. However, our study was limited by its small sample size and the fact that it was conducted at a single institute. Hence, our findings may not be generalizable to the wider population; nonetheless, we can claim that the results favor the AIR band in terms of band removal time. We recommend large follow-up studies so that findings broader enough to be generalizable to the wider population can be attained.

## Conclusions

Based on our findings, the AIR band is more effective in reducing the radial artery compression time, but both devices had insignificant differences between them in terms of RAO in the short term; follow-up studies are required to see if the AIR band is associated with any long-term benefits. Hence, we recommend that further follow-up and large-scale studies be conducted.
